# A new case of *de novo* 6q24.2-q25.2 deletion on paternal chromosome 6 with growth hormone deficiency: a twelve-year follow-up and literature review

**DOI:** 10.1186/s12881-015-0212-z

**Published:** 2015-08-23

**Authors:** Stefano Stagi, Elisabetta Lapi, Marilena Pantaleo, Massimo Carella, Antonio Petracca, Agostina De Crescenzo, Leopoldo Zelante, Andrea Riccio, Maurizio de Martino

**Affiliations:** Endocrine Paediatric Unit, Department of Health Sciences, University of Florence, Anna Meyer Children’s University Hospital, Viale Pieraccini 24, 50139 Florence, Italy; Genetics and Molecular Medicine Unit, Anna Meyer Children’s University Hospital, Florence, Italy; Medical Genetics Unit, IRCCS Casa Sollievo Della Sofferenza Hospital, San Giovanni Rotondo, Italy; Department of Environmental Science, Second University of Naples, Caserta, Italy

**Keywords:** 6q24.2-q25.2 deletion, Short stature, Growth hormone deficiency, Chromosome deletion, Growth failure

## Abstract

**Background:**

Deletions on the distal portion of the long arm of chromosome 6 are relatively uncommon, and only a small number occurs in the paternal copy, causing growth abnormalities. As a result, extensive clinical descriptions are lacking.

**Case presentation:**

We describe a male of Italian descent born at 35 weeks by elective caesarean delivery presenting hypoplastic left colon, bilateral inguinal hernia, dysplastic tricuspid and pulmonary valves, premature ventricular contractions, recurrent otitis media, poor feeding, gastro-oesophageal reflux, bilateral pseudopapilledema, and astigmatism. He also showed particular facial dysmorphisms and postnatal growth failure. Early psychomotor development was mildly delayed. At 3.75 years, he was evaluated for severe short stature (−2.98 SD) and delayed bone age. He showed an insulin-like growth factor 1 concentration (IGF-1) in the low-normal range. Growth hormone stimulation tests showed a low response to clonidine and insulin. Magnetic resonance imaging showed hypophyseal hypoplasia**.** Genetic evaluation by Single Nucleotide Polymorphism arrays showed a *de novo* 6q24.2-q25.2 deletion on paternal chromosome 6.

**Conclusion:**

We confirm that this is a new congenital malformation syndrome associated with a deletion of 6q24.2-q25.2 on paternal chromosome 6. We suggest evaluating the growth hormone axis in children with 6q24.2-q25.2 deletions and growth failure.

## Background

Interstitial and terminal deletions of the long arm of chromosome 6 have been known since 1975 [1] and are relatively uncommon disorders. Just over 100 cases have been reported to date [2–10].

The variability of size and location of specific deletions and the lack of molecular mapping of breakpoints have made it difficult to establish genotype–phenotype correlations [11], even though 6q25 is a preferential location for breakpoints in the 6q terminal deletions [11, 12].

With one notable exception [13], all patients experienced mild to moderate intellectual disability [14]. In addition, deletions involving band 6q25 have a high (63 %) incidence of intrauterine growth retardation (IUGR) [5]. Cryptorchidism appears to be common in patients with middle and terminal deletions [4, 5].

Kumar et al. [13], and Nowaczyk et al. [14] reported six children with *de novo*, paternal, interstitial deletions involving the 6q24.3 region; one of these children was a subject of a previous publication [13]. Some patients had strikingly similar facial features, significant IUGR and postnatal growth retardation with early developmental delay [13, 14]. The third patient had IUGR and normal development [13], which started a debate about whether a new congenital malformation syndrome was identified [13]. While their growth rate may not coincide with a growth chart, there is limited evidence of the causes of growth delay in these children.

We describe herein a new case of *de novo* 6q24.2-q25.2 deletion on paternal chromosome 6 with growth hormone (GH) deficiency. We evaluated the association between the deletion and the patient’s symptoms and followed up with recombinant human growth hormone (rhGH) treatment. Finally, we compared previously published cases with deletions overlapping our patient’s (Table 1) using the Decipher database [15].

## Case presentation

The propositus was the third child of healthy, non-consanguineous Italian parents. The mother was 165 cm and had menarche at 12 years of age. The father was 172 cm and had a normal pubertal development. The target height was 175 cm ± 6 cm (−0.32 SDS). Two sisters of the propositus were growing in the target range.

The patient was born by elective cesarean delivery to a 29-year-old gravida after normal conception and 35 weeks of gestation. The foetus was suspected of IUGR from the 27th week. His birth weight was 1740 g (−1.6 SDS), length was 43.5 cm (−1.1 SDS), and head circumference was 32.2 cm (0.0 SDS). The child’s Apgar score was 7^I^-9^V^.

Prenatal ultrasounds also showed apparent cardiomegaly, which was not confirmed postnatally. Following amniocentesis, the karyotype was found to be 46,XY. Microsatellite analysis of chromosomes 2, 11, and 16 showed biparental inheritance, excluding uniparental paternal disomy.

As a newborn, the propositus was hospitalized for hypoplastic left colon, which caused constipation in the first few months of life.

Early psychomotor development was mildly delayed. He rolled over at 5 months, sat alone and pulled to stand at 10 months, said his first words at 18 months, walked alone at 19 months, and said 25 single words at 2 years of age, but used no sentences.

**Fig. 1:**
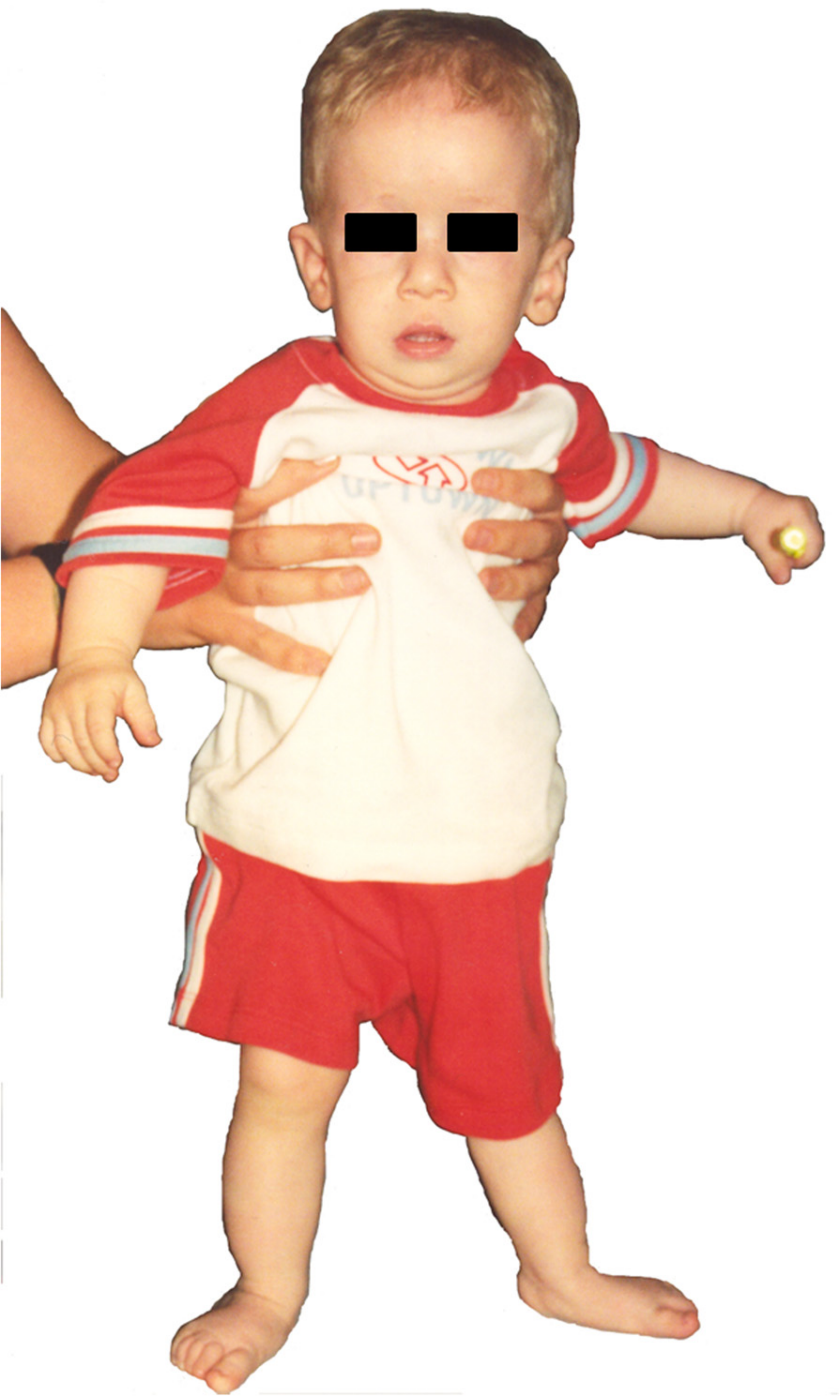
Anteroposterior view of the patient at 12 months of age

He was referred for genetic evaluation at age 13.5 months because of his poor growth. His medical problems included dysplastic tricuspid and pulmonary valves with mild regurgitation, mitral valve prolapse, chronic otitis media, poor feeding, and gastro-oesophageal reflux. Ophthalmoscopic examination showed bilateral pseudopapilledema and hyperopic astigmatism. He showed a relative macrocrania with particular facial features such as prominent forehead, epicanthic folds, upslanting palpebral fissures, large ears and broad nasal bridge (Table 1, Fig. 1). At 2 years of age, he underwent a bilateral inguinal herniotomy.

**Table 1:** Review of main phenotypic characteristics of patients with deletions overlapping 6q24.2-q25.2

Clinical findings	McLeod [2]	Kumar [13]	Sukumar [6]	Sukumar [6]	Sukumar [6]	Meng [4]	Narahara [3]	Bisgaard [19]	Tanteles [20]	Caselli [21]	Nowaczyk [14]	Nowaczyk [14]	Our Case	Total
6q breakpoints	q23-q25	q23.3-24.2	q24.2-q25.1	q25.1-q25.3	q25.1-q26	q24.3-ter	q25-1-q25.3	q25.1-25.3	q24.3-25.2	q24.3-q25.1	q25.1-q25.3	q25.1-q25.3	q24.2-q25.2	13
Parent of origin	NA	father	NA	NA	NA	NA	NA	NA	NA	NA	father	father	father	4/4
Sex (M:F)	M	F	M	M	M	M	M	F	M	F	M	F	M	9/4
Age (yrs.months)	<0.1	3.0	1.0	17	<0.1	0.4	0.7	6.6	2.6	8.2	1.3	1.4	1.-1	-
IUGR/low birth weight	+	+	+	-	-	+	+	-	-	-	+	+	+	8/13
Postnatal growth failure	+	+	+	-	+	+	+	+	-	+	+	+	+	11/13
Microcephaly	+	-	+	+	+	+	+	NA	-	-	-	-	-	7/12
Prominent forehead	-	-	+	+	-	-	-	+	+	-	+	+	+	7/12
Epicanthic folds	+	+/−	+	+	-	+	+	+	-	-	-	-	+	8/13
Downslanting palpebral fissures	-	-	-	+	-	+	-	-	-	+/−	-	-	-	4/13
Upslanting palpebral fissures	-	-	-	-	-	-	+	+	-	-	+	+	+	5/13
Retinal, macular abnormalities	-	ND	-	-	+	ND	ND	ND	-	ND	-	-	-	1/8
Large ears	-	NA	-	+	-	+	NA	-	-	-	-	-	+	3/11
Malformed ears	-	NA	-	+	+	+	+	+	-	+	+	+	-	8/12
Broad nasal bridge	-	+	+	-	-	+	+	-	+	-	-	-	+	6/13
Micrognathia	+	-	-	+	-	+	+	-	-	-	-	-	-	4/13
Abnormal philtrum	-	-	-	-	-	-	-	-	+	+/−	-	-	-	2/13
Microstomia	+	-	NA	NA	NA	NA	+	-	+	-	-	-	-	3/9
Thin lips	-	+	+	-	-	NA	NA	+	-	+	+	+	-	6/11
Congenital heart defect	+°°	-	+^§§^	-	-	+^d^	-	+’	-	+^§^	+°	+^&^	+	8/13
Respiratory distress	-	-	+	-	+	+	NA	-	-	+	-	+	-	5/12
Feeding problems	-	+	-	+	-	-	.	-	+	+	-	-	+	5/12
Cryptorchidism	-		+	-	+	+	-		+				-	4/8
Genital hypoplasia	-	NA	NA	NA	-	NA	-	-	NA	NA	-	-	-	0/7
Short neck	+	+	-	-	-	+	+	NA	-	-	-	-	-	4/12
Chest and trunk asymmetry	-	NA	-	+	-	+	-	NA	-	NA	-	+	-	3/10
Spine abnormalities	+	ND	-	-	-	ND	ND	ND	-	ND	-	-	-	1/8
Foot abnormalities	-	NA	-	+	+	+	-	-	-	+	NA	NA	+	5/10
Abnormal hands, fingers	+	NA	-	+	-	+	+	-	-	+	+	NA	+	7/11
Joint laxity	-	NA	-	-	-	NA	NA	NA	+	NA	NA	NA	+	2/6
Hypotonia	-	-	+	-	-	+	+	NA	+	NA	NA	+	+	6/10
Skin abnormalities	+	NA	-	-	-	NA	NA	NA	+	NA	NA	NA	-	2/6
Seizures	-	-	-	+	-	+	.	-	-	-	-	-	+	3/13
MRI/TC abnormalities	ND	ND	ND	-	+^a^	+^b^	ND	ND	ND	-	-	-	+^c^	3/7
Developmental delay	NA	-	+	+	NA	+	+	+	-	+	+	+	+	9/11
Other	+^1, 2^					+^1^							+^3^	

*NA* not available, *ND* Not determinable

^a^Agenesis of corpus callosum and temporal and occipital enlargement; ^b^agenesis of corpus callosum; ^c^pituitary hypoplasia with a cyst in the pars intermedia; ^d^ventricular septal defect and patent ductus arteriosus; ‘ventricular septal defect; °dysplastic tricuspid and pulmonary valves, premature ventricular contractions; °°systolic murmur without echocardiographic evaluation; +^§§^tricuspid regurgitation; +^1^Imperforate anus; +^2^sacrum lipoma; +^3^hypoplastic left colon and inguinal hernia

+& secundum atrial septal defect

+§ atrial septal defect

**Fig. 2:**
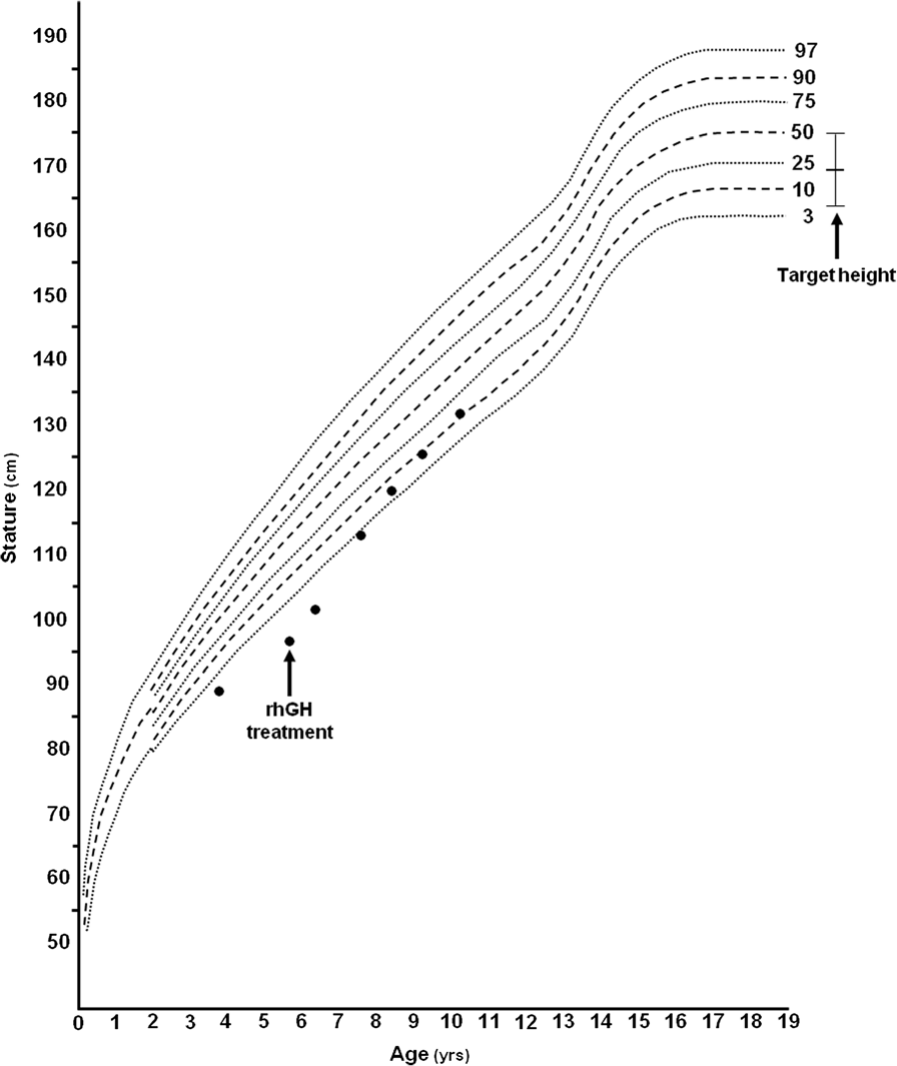
Growth charts of the patient. The arrows indicate the onset of growth hormone therapy and the target height (TH)

At 3 years 9 months of age, his height was 88.2 cm (−2.98 SDS), his weight was 11.500 Kg (−3.34 SDS), his body mass index (BMI) was 14.78 (−0.81 SDS), and his occipitofrontal circumference was 50 cm (0.33 SDS) (Fig. 2). A bone age evaluation revealed a 28-month delay. Extensive biochemical and metabolic examinations did not reveal abnormalities. The patient showed only slight hyperthyreotropinemia (TSH 5.49 mIU/L; normal range: 0.61–4.0 mIU/L). The plasma concentrations of insulin-like growth factor 1 (IGF-1) and IGF binding protein-3 (IGFBP-3) were in the lower part of the normal range: 58 ng/ml (normal range 44–221 ng/ml) and 1.4 μg/ml (normal range 1.0–4.7 μg/ml) for IGF-1 and IGFBP-3, respectively.

At 5 years 8 months of age, his height was 96 cm (−3.66 SDS), his weight was 13.500 kg (−3.67 SDS), and his BMI was 14.65 (−0.91) (Fig. 2). A blood examination confirmed slight hyperthyreotropinemia (TSH 5.1 mIU/L) with normal FT_4_. The plasma concentrations of IGF-1 and IGFBP-3 were in the lower part of the normal range for age and sex: 63 ng/ml (normal range: 54–228 ng/ml) and 1.3 μg/ml (normal range: 1.3–5.6 μg/ml) for IGF-1 and IGFBP-3, respectively.

At 6 yrs of age, an echocardiogram showed a bicuspid aortic valve. A magnetic resonance imaging (MRI) scan of the brain showed peritrigonal T2 white matter hyperintensity, in the absence of midline anomalies or anatomic defects of the central nervous system. The hypothalamic-hypophyseal region also showed pituitary hypoplasia with a cyst in the pars intermedia**.** Neuropsychological evaluation showed that deficits persisted, particularly in auditory-language association, auditory pattern recognition, and interhemispheric integration.

Because of the persistent and severe growth failure, the child was evaluated with GH stimulation tests, which showed a low response after clonidine (GH peaked at 4.46 μg/l) and insulin (GH peaked at 3.14 μg/l) tests. Consequently, GH therapy (0.23 mg/kg/wk) was started.

The child experienced a good response to GH therapy (Fig. 2). At 7 years 7 months of age, his height was 113.5 cm (−2.20 SDS), his weight was 19.800 kg (−1.81 SDS), his BMI was 15.37 (−0.66 SDS), and his Tanner pubertal development stages were at G1 (genital development), PH1 (pubic hair), and AH1 (auxiliary hair), with a bilateral testicular volume of 2.5 ml.

His pubertal development started normally: at 11 years 4 months, his height was 137.6 cm (−1.27 SDS), his weight was 31.000 kg (−1.38 SDS), his BMI was 16.4 (−1.04 SDS), and his pubertal staging was: testicular volume of 5–6 ml bilaterally, PH2, and AH2 (Fig. 2). The patient showed asymmetry in his body proportion, with a 1.30 ratio between the upper and lower segment.

### Genetic analysis

**Fig. 3:**
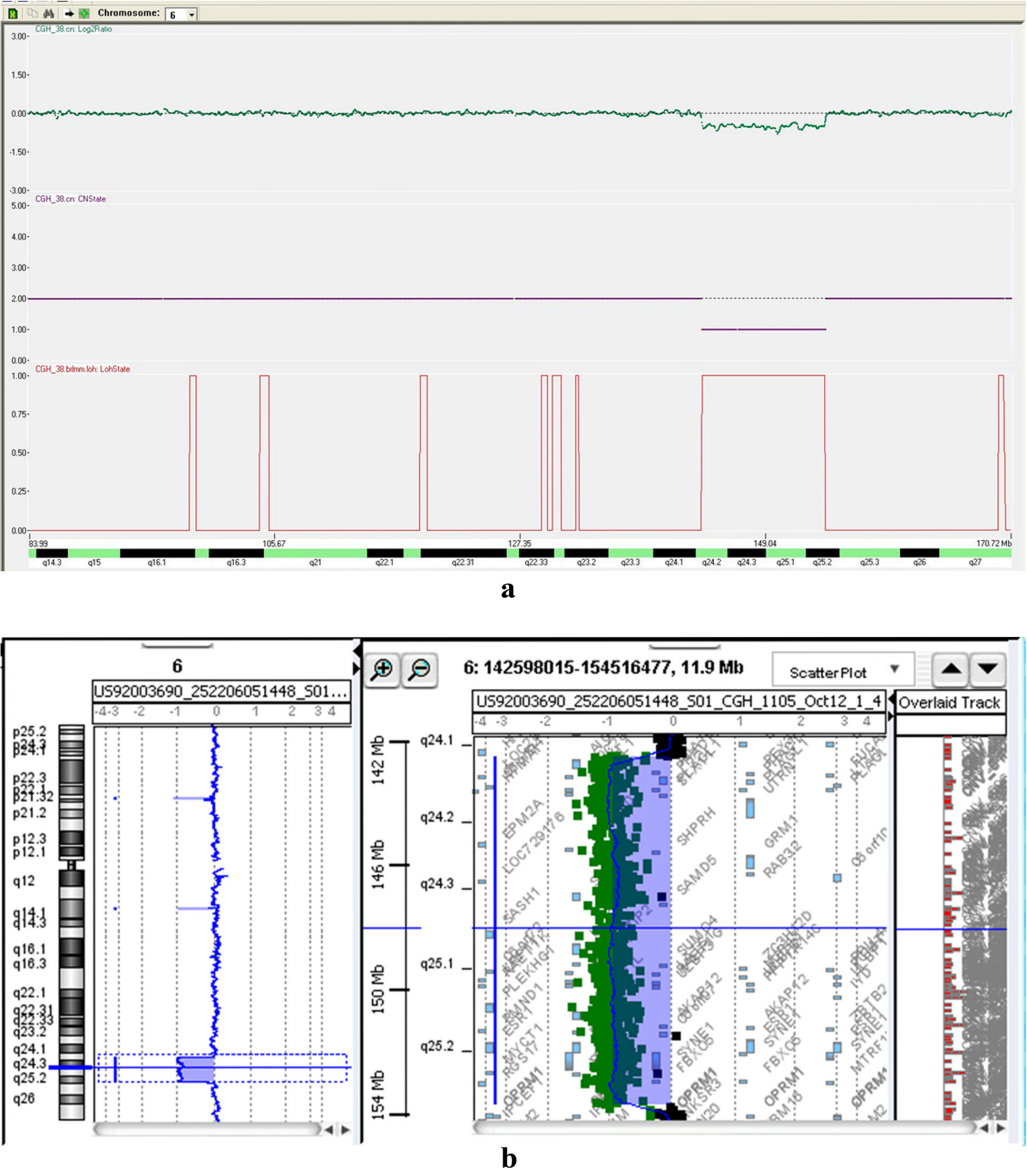
Single Nucleotide Polymorphism (SNP) arrays (Fig. 3**a**) and array CGH with high resolution (Fig. 3**b**) showing a *de novo* deletion of 11Mb 6q24.2-q25.2

**Fig. 4:**
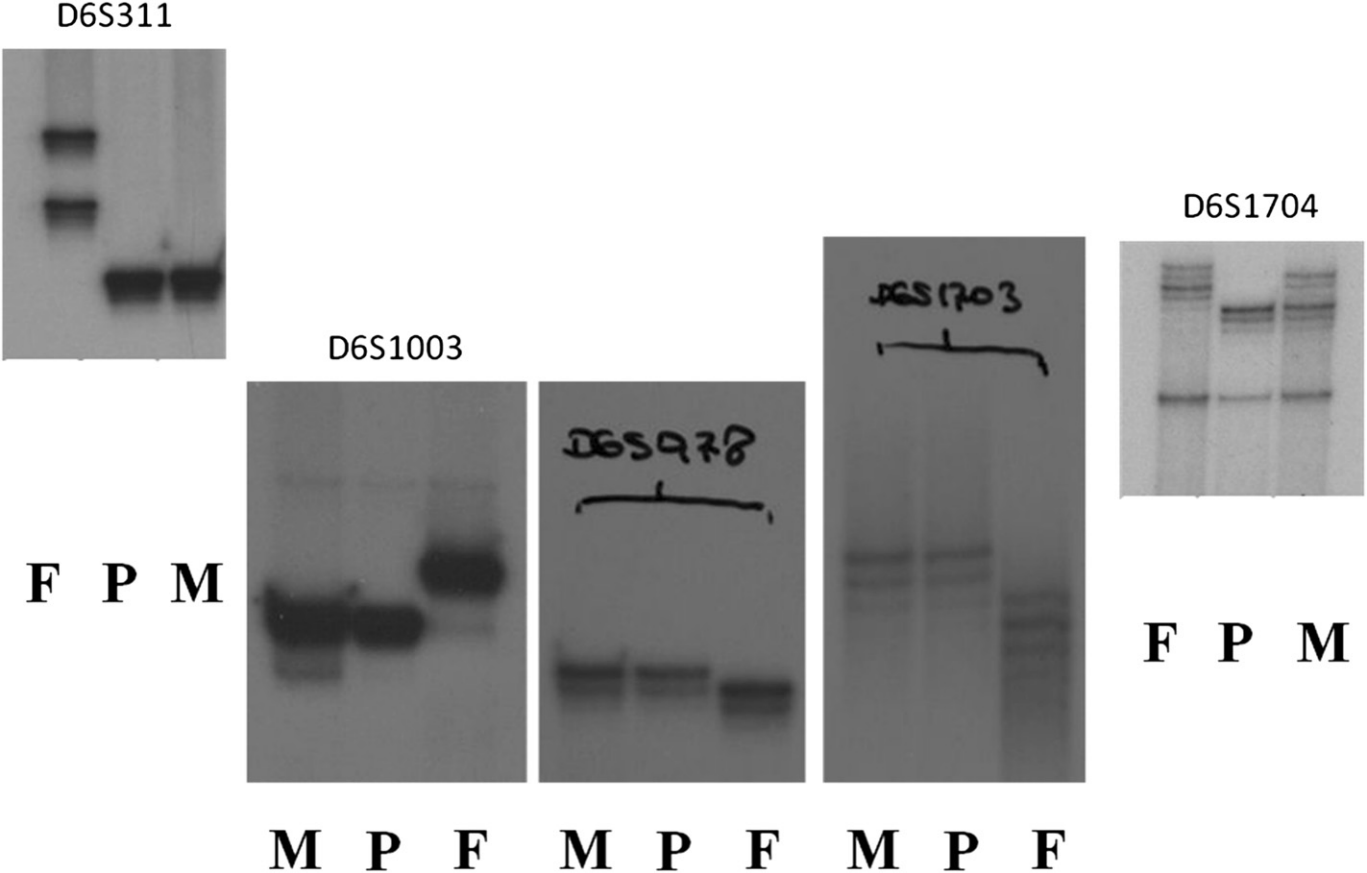
Short Tandem Repeat (STR) analysis demonstrated that deletion occurred on paternal chromosome 6. M = mother; P = proband; F = father

Molecular karyotyping was also performed by array-CGH on the proband’s DNA using an Agilent 180 K array platform with a resolution of approximately 40 kb. Based on the physical mapping positions of the Feb 2009 Assembly (GRCh37/hg19) of the UCSC Genome Browser, this analysis showed a deletion of approximately 10,741 Mbp that involved the 6q24.2q25.2 region, with the breakpoint falling between 143,297,976 bp (first deleted oligomer) and 154,039,064 bp (last deleted oligomer) (Fig. 3a). However, genetic evaluation by single nucleotide polymorphism (SNP) arrays (GeneChip Human Mapping 500 k, Affymetrix) showed a deletion of 11 Mb within the 6q24.2-q25.2 region on paternally inherited chromosome 6 (Fig. 3b). Short Tandem Repeat (STR) analysis demonstrated that the deletion occurred on the paternal 6 chromosome (Fig. 4; see supplement). Paternal sample analysis excluded a balanced insertional translocation.

### Discussion

We described a patient with interstitial deletion of the chromosomal region 6q24.2-q25.2 on paternal chromosome 6, consistent with the observations of Kumar et al. [13] and Nowaczyk et al. [14], who also found a paternal deletion.

Interstitial and terminal deletions of chromosome 6q are rare disorders, commonly associated with intellectual disability, dysmorphic features, growth failure, and medical complications [2, 5, 11].

Paternal interstitial deletions at 6q24.2-q25.2 are rarely described, but some patients can share significant IUGR and postnatal growth retardation, redundant skin, joint laxity, and strikingly similar facial features, associated with early developmental delay [13, 14]. We propose a new congenital malformation syndrome associated with 6q24.2-q25.2 on paternal chromosome 6 [14]. Other patients showed IUGR but developed normally postnatally [13].

Our case report strongly suggests that an impairment of the GH-IGF-1 axis is a possible cause of short stature in this syndrome. Therefore, these patients should be tested for GH secretion.

Interestingly, Nowaczyk et al. reported that one in three patients with paternal deletion of 6q24.3 showed reduced IGF-1 and IGFBP-3 secretion but subnormal GH secretion after GH provocative testing (11.2 and 6.3 ng/ml, respectively) [14]. In this patient, an MRI scan of the brain appeared normal [14]. However, a trial of rhGH therapy was started at age 3.0 years, as his height was 4.0 SD below average, and an excellent response was experienced, increasing height to 1.4 SD below average [14]. The response to GH treatment was good in our patient, showing that GH treatment could also be considered to improve the prognosis with respect to stature in patients with 6q24.2-q25.2 paternal interstitial deletions. Nevertheless, further studies are required to confirm the characteristics of the GH-IGF-1 axis in these patients.

A 6q24.3 deletion of paternal origin is frequently found with *PLAG1* (*Pleomorphic Adenoma Gene 1*; OMIM *603044) or *ZAC1* (the zinc finger gene involved in apoptosis), a gene member of the network of coregulated genes comprising other imprinted genes involved in the control of embryonic growth [16]. *PLAGL1* is an imprinted gene, paternally expressed. Generally, in mouse inactivation of the maternally repressed Plagl1 transcription factor results in intrauterine growth restriction, altered bone formation, and neonatal lethality [16]. This aspect is very interesting, because patients with paternal deletions of 6q24.2-q25.2 showed IUGR, early developmental delay, and a typical facial appearance, suggesting that the genetic basis for this syndrome is located within the deleted 1 Mb region. Nevertheless, recent data seem to suggest that methylation of *PLAGL1* may be associated with foetal and post-natal weight and BMI but not with length [17].

Our 6q24.2-25.2 deletion partially overlaps those reported in Decipher [15], which spare from 3,61 Mb to 8,52 Mb. In nine reported cases (four males and four females and one case with unknown chromosomal sex) the age, at initial presentation, varies from <1 to 8 years (median age 4 years) and growth delay/short stature is a constant reported feature of the phenotype. The deletion is defined *de novo* in one female case, but parent of origin is not determined; the inheritance is unknown in other three cases, while in two males is inherited from a non specified parent. Interestingly in three male cases the deletion is also present and constitutional in their fathers, two of which are referred with similar phenotype to child, i.e. short stature or growth delay, delayed speech and language development. In our case the deletion has occurred on paternal chromosome 6. In some patients, *PLAGL1* gene is not included in the deletions, while in other patients with more centromeric 6q deletions, comprising *PLAGL1* gene, growth impairment is not described as a clinical feature. Anyway the role of this gene in human growth process, remains to be elucidated.

Growth deviations are a common complication in imprinting disorders. Imprinted genes are not bi-allelically expressed, and diseases arise when an individual inherits two copies of a chromosome that contains imprinted genes from one parent (uniparental disomy) [18].

Nevertheless, our patient also showed pituitary hypoplasia as a possible cause of GH deficiency. In fact, some patients with 6q deletions may show congenital midline abnormalities, such as undescended testicles or very small genitalia, heart defects (ventricular septal defects, atrioventricular canal, atrial septal defect, tetralogy of Fallot, etc.), imperforate anus, diaphragmatic hernia, cleft palate, and umbilical hernia [6].

## Conclusions

In conclusion, we report a child with a remarkable facial features, a history of IUGR and postnatal growth failure due to GH deficiency, disproportionate short stature, and minimal developmental delay associated with a 6q24.3 deletion of paternal origin. For the first time, we report the presence of GH deficiency and a long-term follow-up, hypothesizing that dysregulation of the GH-IGF-1 axis may be a frequent characteristic of this syndrome. Therefore, we suggest evaluating the GH axis in children with 6q24.3 deletions and growth failure. Identification of imprinted genes or further defining the expression of genes in the region of deletion may lead to the identification of specific genes important in the regulation of growth.

## Consent

The parents of the patient provided written informed consent for publication of this Case Report and any accompanying images. A copy of the written consent is available for review by the Editor of this Journal. This study was approved by the Anna Meyer Children’s Hospital Ethics Committee.

## Abbreviations

GH: Growth hormone
IUGR: Intrauterine growth retardation
